# Discogenic Lumbar Disease

**DOI:** 10.1155/2012/351714

**Published:** 2012-12-03

**Authors:** Brian R. Subach, Thomas C. Schuler, Mark R. McLaughlin, Paul J. Slosar, Christopher H. Comey, Najeeb M. Thomas

**Affiliations:** ^1^Virginia Spine Institute, 1831 Wiehle Avenue, Suite 200, Reston, VA 20878, USA; ^2^Princeton Brain and Spine Care, 1203 Langhorne-Newtown Road, Suite 138, Langhorne, PA 19047, USA; ^3^Spine Care Medical Group, San Francisco Spine Institute, 1850 Sullivan Avenue, Daly City, CA 94015, USA; ^4^New England Neurosurgical Associates, LLC, 300 Carew Street, Suite One, Springfield, MA 01104, USA; ^5^Southern Brain & Spine, 4228 Houma Boulevard, Suite 510, Metairie, LA 70006, USA

The treatment of discogenic lumbar disease is a major challenge faced by physicians throughout the world. This condition affects many patients and will inevitably become more prevalent with a rapidly aging population. Disc degeneration tends to increase rapidly with age so that 10% of 50-year-old and 60% of 70-year-old discs are severely degenerated [1]. The current special issue explores several crucial angles related to the pathology, diagnosis, and treatment of discogenic lumbar disease.

The mechanism of lumbar disc disease is elucidated in an article by V. K. Goel et al. which outlines the molecular processes involved in disc degeneration and the physical and chemical changes reducing disc integrity. The diagnosis of this condition is extensively explored in two related articles. The first article by A. C. Breen et al. *“Measurement of intervertebral motion using quantitative fluoroscopy: report of an international forum and proposal for use in the assessment of degenerative disc disease in the lumbar spine”* presents the case for using quantitative fluoroscopy application to the measurement of intervertebral motions and degenerative disc diagnosis. In the second article, M. W. Hasz provides a review of the diagnostic procedures for degenerative disc disease. He also provides a succinct explanation of how radiography, computed tomography, magnetic resonance, and discography are utilized in degenerative disc disease diagnosis.

Disc disease treatment is extensively addressed in four articles. The articles by D. Kok et al. and by L. Marchi et al. explore the application of interbody fusion as treatment for severely degenerated discs. V. Popov and D. G. Anderson provide an insightful review of treatments for lumbar disc degeneration and the application of ipsilateral and bilateral decompression with a tubular retractor system under microscopy. D. Drazin et al. provide a review of stem cell injection therapy for the intervertebral disc. 

Evaluation of the outcomes of patients with lumbar disc disease is critical to the assessment of treatment efficacy. C. Lozano-Alvarez et al. describe the use of the Core Outcome Measures Index (COMI) in daily clinical practice for assessing patients with degenerative lumbar disease. 

The current issue covers disc disease from several angles. Our intention is to provide a resource which can enlighten readers as to the mechanism, diagnosis, and the present state of intervertebral disc disease therapy and its treatment advances.

*Brian R. Subach* *Thomas C. Schuler* *Mark R. McLaughlin* *Paul J. Slosar* *Christopher H. Comey* *Najeeb M. Thomas*
